# Incidence of Cefotaxime‐Resistant *Haemophilus influenzae* Is Linked to Regional Consumption of Oral Aminopenicillins and Parenteral Third‐Generation Cephalosporins in Norway

**DOI:** 10.1111/apm.70201

**Published:** 2026-04-06

**Authors:** Dagfinn Skaare, Gunnar Skov Simonsen

**Affiliations:** ^1^ Department of Infection Prevention and Control Vestfold Hospital Trust Tønsberg Vestfold Norway; ^2^ Department of Microbiology Vestfold Hospital Trust Tønsberg Vestfold Norway; ^3^ Department of Microbiology and Infection Control University Hospital of North Tromsø Troms Norway; ^4^ Faculty of Health Sciences UiT the Arctic University of Norway Tromsø Troms Norway

**Keywords:** antibiotic resistance, antibiotic stewardship, Beta‐lactams, prescription behaviour, rational use

## Abstract

The emergence of cefotaxime‐resistant *Haemophilus influenzae* (CRHI) in Norway during the 2000s coincided with increased use of oral aminopenicillins and parenteral third‐generation cephalosporins. This study examined associations between beta‐lactam consumption and CRHI incidence. Surveillance data on antibiotic usage across Norwegian regions (2013–2017) and CRHI incidence in hospitalised patients (January 2013–March 2018) from a previous study were analysed. Correlations were calculated between average consumption rates for aminopenicillins and third‐generation cephalosporins, and average CRHI incidence rates. Regional aminopenicillin prescription and hospital sales of third‐generation cephalosporins varied substantially. CRHI incidence ranged from 0.8 to 3.2 cases per million inhabitants per year and was significantly associated with use of oral aminopenicillins in primary care (*r* = 0.88, *p* = 0.022), hospital use of third‐generation cephalosporins (*r* = 0.88, *p* = 0.019), and both variables combined (*r* = 0.89, *p* = 0.007). The consumption variables also showed significant covariation (*r* = 0.88, *p* = 0.020). Regional variation in consumption of oral aminopenicillins and parenteral third‐generation cephalosporins is linked to CRHI incidence in Norway. These findings strengthen the evidence base for avoiding unnecessary use of these agents and underscore the need for coordinated antibiotic stewardship across healthcare sectors to limit resistance development.

## 1 Introduction

*Haemophilus influenzae* commonly colonises the human respiratory tract in all age groups [1]. Person‐to‐person transmission is frequent and may lead to infections ranging from mild upper respiratory tract illness to life‐threatening invasive disease [1, 2, 3]. *H. influenzae* is the leading bacterial cause of exacerbations in chronic obstructive pulmonary disease (COPD) [4], and recent investigations suggest it is also the most frequent cause of non‐bacteraemic community‐acquired pneumonia [5].

Vaccination against encapsulated *H. influenzae* serotype b (Hib) has markedly reduced the incidence of invasive disease in young children but does not protect against other serotypes or non‐typeable *H. influenzae* (NTHi) [3]. As a major global driver of antibiotic use and resistance, *H. influenzae* is included on the World Health Organisation (WHO) bacterial pathogen priority list to guide research, development and public health strategies against antimicrobial resistance [6].

In Norway, narrow‐spectrum penicillins (phenoxymethylpenicillin or benzylpenicillin) are recommended as first‐line empiric therapy for uncomplicated community‐acquired respiratory tract infections, while aminopenicillins (amoxicillin or ampicillin) are preferred for COPD exacerbations because *H. influenzae* exhibits intrinsically reduced susceptibility to narrow‐spectrum penicillins [7, 8]. To address resistance mediated by beta‐lactamase production and/or alterations in penicillin‐binding protein 3 (PBP3) [2, 9] – which affects 15%–20% of *H. influenzae* isolates in Norway [10] – cefotaxime, a third‐generation cephalosporin, is recommended for empiric treatment of severe community‐acquired pneumonia of unknown aetiology and COPD exacerbations with severe respiratory failure [7].

Given the potential consequences of therapy failure in such cases, the emergence and spread of cefotaxime‐resistant *H. influenzae* (CRHI) is a global concern [11]. Cefotaxime‐resistant strains, as defined by the European Committee on Antimicrobial Susceptibility Testing (EUCAST) (https://www.eucast.org/breakpoints), typically have amino acid substitutions in at least two key positions near the active site in the transpeptidase region of PBP3, encoded by the *ftsI* gene [9, 11, 12]. PBP3 is the main target for aminopenicillins and third‐generation cephalosporins, and key substitutions confer increased MIC for both categories (cross‐resistance). However, EUCAST breakpoints categorise most strains with only one key substitution as susceptible to cefotaxime, and approximately half as resistant to aminopenicillins [9, 12]. PBP3 substitutions are acquired through point mutations and transformation with *ftsI* sequences from resistant strains within genus *Haemophilus*, including the commensal species *H. haemolyticus* and *H. parainfluenzae* [2, 11, 13]. Cefotaxime resistance may occur from a single transformation event within a single host or evolve gradually, involving multiple hosts across age groups, health care sectors, and borders [11].

CRHI are particularly prevalent in some Asian regions [12, 14] but are also increasing in Europe, partly due to expansion of international clones [11, 15]. The sharp rise in CRHI prevalence in Japan during the late 1990s and early 2000s was linked to widespread use of oral cephalosporins [12]. In Norway, the emergence and clonal dissemination of domestic and imported CRHI strains coincided with a 75% increase in oral amoxicillin use and a 200% increase in cefotaxime use between 2000 and 2012, whereas the use of narrow‐spectrum penicillins was relatively stable during the same period [10, 11].

While supported by experimental studies [16, 17], the association between use of beta‐lactam antibiotics and CRHI incidence at the population level has to our knowledge not previously been investigated. The aim of this study was therefore to examine whether the use of aminopenicillins and third‐generation cephalosporins influences the incidence of CRHI in Norway.

## 2 Materials and Methods

Six geographical regions were defined based on the catchment areas of the South‐Eastern, Western, Central, and Northern regional health authorities (https://sml.snl.no/regionalt_helseforetak). To balance population sizes, the South‐Eastern region was divided into Southern, Eastern, and Capital regions (Table 1). Population data were obtained from Statistics Norway (https://www.ssb.no).

**TABLE 1:** Consumption of aminopenicillins and third‐generation cephalosporins and incidence of cefotaxime‐resistant *H. influenzae* (CRHI) in Norway.

Region^a^	Proportion of the population (%)^b^	Antibiotic consumption^c^						CRHI incidence^d^	
		Community prescription^e^		Hospital sales^f^					
		Amoxicillin	Amoxicillin‐clavulanic acid	Amoxicillin	Ampicillin	Third‐generation cephalosporins	Ratio^g^	Number of isolates	Incidence rate
Eastern	29.7	0.75	0.01	0.03	0.03	0.13	1.9	20	2.5
Western	21.0	0.65	0.01	0.04	0.03	0.12	1.8	8	1.4
Southern	13.8	0.81	0.01	0.03	0.03	0.15	2.3	12	3.2
Central	13.7	0.65	0.01	0.03	0.04	0.12	1.6	9	2.4
Capital	12.5	0.75	0.01	0.03	0.03	0.14	2.3	9	2.6
Northern	9.3	0.62	0.00	0.05	0.04	0.09	1.0	2	0.8
Total	100.0	0.71	0.01	0.04	0.03	0.13	1.8	60	2.2

^a^ Eastern region, Akershus, Buskerud, Innlandet, and Østfold counties; Western region, Rogaland and Vestland counties; Southern region, Agder, Telemark, and Vestfold counties; Central region, Møre og Romsdal and Trøndelag counties; Capital region, Oslo County; Northern region, Finnmark, Nordland, and Troms counties (https://snl.no/fylke).

^b^ Calculated from total number of inhabitant years (2013–2017). Data from Statistics Norway (www.ssb.no).

^c^ Average consumption rates (2013–2017) expressed as defined daily doses (DDD) per 1000 inhabitants per day (DID).

^d^ CRHI incidence among hospitalised patients (January 2013–March 2018). Data from [11]. Average CRHI incidence rates expressed as cases per million inhabitants per year (CIY).

^e^ Based on data from the Norwegian Prescription Database (https://www.norpd.no).

^f^ Based on data from Hospital Pharmacies Drug Statistics.

^g^ Use of third‐generation cephalosporins to combined use of amoxicillin and ampicillin.

Data on community prescription of oral aminopenicillins (amoxicillin and amoxicillin‐clavulanic acid, ATC codes J01CA04 and J01CR02) were retrieved from the Norwegian Prescription Database (https://www.norpd.no). Hospital sales of aminopenicillins (amoxicillin, amoxicillin‐clavulanic acid, and J01CA01 ampicillin) and third‐generation cephalosporins (J01DD, including J01DD01 cefotaxime and J01DD04 ceftriaxone) were obtained from the Hospital Pharmacies' Drug Statistics and used as a measure of hospital prescription. Average community and hospital consumption rates expressed as defined daily doses (DDD) per 1000 inhabitants per day (DID) (2013–2017) were calculated for each region and nationally (Table 1). Temporal trends were not calculated.

Calculation of average national and regional CRHI incidence rates (cases per million inhabitants per year, CIY) (Table 1) was based on anonymised data from a previously published molecular epidemiologic study of CRHI in Norway and Sweden (2006–2018) [11]. The study was initiated because EUCAST previously classified CRHI as an exceptional phenotype and recommended confirmation at a reference or expert laboratory [18]. In Norway, this was provided at Vestfold Hospital Trust until March 2018, when the service was terminated because cefotaxime resistance was no longer considered exceptional in *H. influenzae* . Resistance was confirmed using broth microdilution [11], with interpretation of MIC values in line with EUCAST guidelines (susceptible ≤ 0.125 mg/L, resistant > 0.125 mg/L) (https://www.eucast.org/breakpoints).

To reduce collection bias, only CRHI from hospitalised patients (first isolate from each patient) was included in the present study, as testing of cefotaxime is optional for primary care isolates in Norway [19]. For the same reason, the study period was limited to 2013 onwards, when all 16 Norwegian hospital laboratories submitted isolates, and at least half contributed annually. The 60 CRHI isolates included were assumed to reflect the true incidence in Norway.

Pearson correlation coefficients (*r*) and *p* values were calculated using SPSS to assess associations between regional antibiotic consumption and CRHI incidence (Table 1). Correlations between consumption variables were also analysed to evaluate suitability for multivariable linear regression. Statistical significance was defined as *p* < 0.05.

## 3 Results

Average community prescription of oral aminopenicillins (with or without clavulanic acid) was 0.71 DID overall, ranging from 0.62 DID in the Northern region to 0.81 DID in the Southern region (Table 1). Amoxicillin (without clavulanic acid) accounted for 99.0% of the total volume.

Average hospital use of third‐generation cephalosporins was 0.13 DID overall, with regional variation from 0.09 DID in the Northern region to 0.15 DID in the Southern region. Cefotaxime and ceftriaxone represented 81.1% and 14.5%, respectively, of the total volume. Combined hospital sales of amoxicillin and ampicillin were 0.07 DID, ranging from 0.06 DID in the Capital region to 0.09 DID in the Northern region. Amoxicillin accounted for 51.1% of this volume.

**FIGURE 1:**
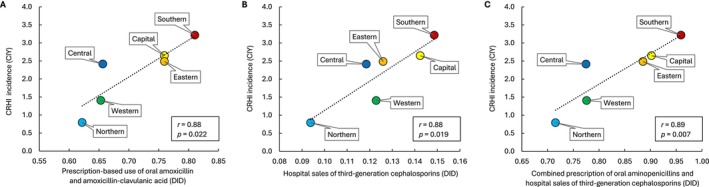
Correlation between average consumption of selected broad‐spectrum beta‐lactam antibiotics (2013–2017) and incidence of cefotaxime‐resistant *Haemophilus influenzae* (CRHI) in six geographic regions in Norway (hospital patients, January 2013–March 2018). (A) Combined prescription‐based use of oral amoxicillin and amoxicillin‐clavulanic acid. (B) Hospital sales of third‐generation cephalosporins. DID, defined daily doses (DDD) per 1000 inhabitants per day. (C) Combined prescription of oral aminopenicillins (amoxicillin and amoxicillin‐clavulanic acid) and hospital sales of third‐generation cephalosporins. CIY, cases per million inhabitants per year.

Average overall incidence rate of CRHI among hospitalised patients was 2.2 CIY (Table 1). Average regional incidence rates ranged from 0.8 CIY in the Northern region to 3.2 CIY in the Southern region. CRHI occurrence was strongly and significantly associated with community prescription of oral aminopenicillins (*r* = 0.88, *p* = 0.022) (Figure 1A), hospital use of third‐generation cephalosporins (*r* = 0.88, *p* = 0.019) (Figure 1B), and the two consumption variables combined (*r* = 0.89, *p* = 0.007) (Figure 1C).

A strong association was also observed between CRHI incidence and cefotaxime use alone, although this correlation was not statistically significant (*r* = 0.73, *p* = 0.103) (not shown). Conversely, there was a strong negative, non‐significant correlation between CRHI incidence and hospital use of amoxicillin and ampicillin (*r* = −0.76, *p* = 0.077) (not shown).

**FIGURE 2:**
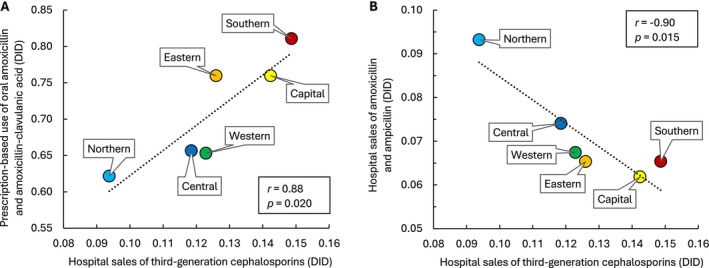
Correlation between average hospital sales of third‐generation cephalosporins and consumption of aminopenicillins in six geographic regions in Norway (2013–2017). (A) Combined prescription‐based use of oral amoxicillin and amoxicillin‐clavulanic acid. (B) Combined hospital sales of amoxicillin and ampicillin. DID, defined daily doses (DDD) per 1000 inhabitants per day.

Correlation analyses revealed multicollinearity between different consumption variables. There was a strong, statistically significant positive correlation between combined prescription of amoxicillin and amoxicillin‐clavulanic acid in primary care and hospital sales of third‐generation cephalosporins (*r* = 0.88, *p* = 0.020) (Figure 2A). Hospital sales of third‐generation cephalosporins and aminopenicillins were strongly and significantly negatively correlated (*r* = −0.90, *p* = 0.015) (Figure 2B), with regional ratios ranging from 1.0 in the Northern region to 2.3 in the Capital and Southern regions (Table 1).

Because the consumption variables were not independent, multivariable regression analysis was not performed.

## 4 Discussion

The link between antibiotic use and resistance is evident from a Darwinian perspective, yet numerous factors complicate the detection of correlations at the population level. It has therefore been argued that when correlations are shown, they are “almost certainly of major importance” and suggest that reducing the use of correlated antibiotics will reduce resistance [20].

In this study, a strong, statistically significant positive correlation was identified between consumption of two broad‐spectrum beta‐lactam categories–oral aminopenicillins and parenteral third‐generation cephalosporins–and the incidence of cefotaxime‐resistant *H. influenzae* (CRHI) in Norway. A role of these drugs in the development and/or spread of CRHI strains is plausible. As perhaps the most frequent bacterial cause of infections in the respiratory tract and adjacent niches–although often not acknowledged in pneumonia and COPD exacerbations due to diagnostic limitations [2, 5] – and a common coloniser in all age groups [1], including patients receiving treatment for infections caused by other organisms, *H. influenzae* is frequently exposed to both antibiotic categories. The same goes for closely related commensals involved in resistance development [1, 13]. Also of note is the ability of *H. influenzae* to form protective biofilm, where the bacteria may survive beta‐lactam treatment despite in vitro susceptibility [4]. A contribution of broad‐spectrum beta‐lactams to development of cefotaxime resistance is further supported by mechanisms of action and resistance [9, 11] and by experimental studies [16, 17]. In addition, treatment of a CRHI carrier with aminopenicillins or third‐generation cephalosporins – irrespective of whether the strain is domestic or imported [11] – would likely increase the bacterial load and the probability of infection and transmission to close contacts. Taken together, our findings strengthen the evidence base for avoiding unnecessary use of these drugs.

CRHI incidence was significantly correlated with use both in primary care and hospitals (Figure 1A,B), but the strongest correlation was obtained by combining the two consumption variables (Figure 1C). This may in part be explained by the combined selective pressure on CRHI carriers as described above. A likely additional explanatory model is that the selective pressure exerted by oral aminopenicillins contributes to a higher prevalence of low‐level resistant *H. influenzae* with a single key PBP3 substitution, and that such strains have a higher probability of developing cefotaxime resistance through acquisition of additional substitutions when exposed to third‐generation cephalosporins. These findings underscore the need for coordinated antibiotic stewardship across healthcare sectors to limit resistance development.

There was also a strong, statistically significant positive correlation between prescription‐based use of oral aminopenicillins and hospital use of parenteral third‐generation cephalosporins (Figure 2A). As this represented multicollinearity, the two explanatory variables could not be combined in a multivariable regression model, and their relative contribution to CRHI incidence could not be compared. However, combined prescription of amoxicillin and amoxicillin‐clavulanic acid in primary care was more than five times higher than hospital use of third‐generation cephalosporins (Table 1), suggesting that even small changes in the prescribing behavior of general practitioners could substantially reduce the resistance‐driving effect of oral aminopenicillins in Norway.

A possible factor contributing to the observed covariation between community and hospital consumption of broad‐spectrum beta‐lactams is variation in COPD prevalence, which is highest in urban areas in southern and eastern Norway [21]. High COPD prevalence may justify greater use of oral aminopenicillins and third‐generation cephalosporins, which are key agents for treating COPD exacerbations in Norway [7, 8].

In addition to demographic differences such as age and sex distribution [10], less studied factors may contribute to regional variation in antibiotic use. A Norwegian study found that community prescribing of antibiotics decreased with increasing latitude, although the effect was less pronounced in municipalities with high population density [22]. This pattern may reflect better access to healthcare services–including pharmacies–in urban areas, but other contributing factors cannot be excluded. For example, the perception that “only the best is good enough” may be more common in cities, where access to services generally is greater than in rural areas.

As hospital physicians are part of the same communities, such attitudes may also influence prescribing practices in hospitals. This interpretation is supported by the strong, statistically significant negative correlation between hospital use of third‐generation cephalosporins and aminopenicillins, suggesting unwarranted overuse of third‐generation cephalosporins in several regions (Figure 2B). Norwegian guidelines recommend aminopenicillins–alone or combined with gentamicin–as standard empirical therapy for common clinical scenarios such as infectious COPD exacerbations and sepsis with suspected urinary tract or abdominal origin, while third‐generation cephalosporins are reserved for patients with severe infection or contraindications to penicillins or aminoglycosides [7]. A possible explanation for the higher ratio of third‐generation cephalosporin to aminopenicillin use in Capital and Southern regions compared to the Northern region is that geographically dependent sociocultural differences influence decision‐making in hospitals, especially in cases where severity assessment and perceived risk of gentamicin‐related adverse effects play a key role in determining empirical antibiotic therapy [23].

Norwegian studies have identified several factors influencing prescribing behaviour among general practitioners [24] and hospital physicians [25], but have not addressed the role of geographical or sociocultural differences. This study illustrates the need for further research on such barriers to improving antibiotic prescribing in Norway [26]. Antibiotic stewardship will always face challenges, but better knowledge of where these challenges are greatest and which counterforces exist is essential for planning and implementing targeted interventions.

Restrictive use of third‐generation cephalosporins is firmly established within the WHO AWaRe (*Access*‐*Watch*‐*Reserve*) framework, where these agents are classified as *Watch* [27]. In contrast, amoxicillin is listed in the *Access* group, comprising first‐ or second‐choice empiric treatment options “showing lower resistance potential than antibiotics in the other groups”. Of note is that Scandinavian guidelines for managing lower respiratory tract infections in primary care differ from international guidelines by recommending phenoxymethylpenicillin as first‐line empiric therapy, while reserving amoxicillin for COPD exacerbations [8, 28, 29, 30]. Restrictive use of amoxicillin is partly supported by a randomised, placebo‐controlled trial demonstrating a significant increase in oral streptococci resistant to penicillin and amoxicillin during amoxicillin treatment [31]. However, this difference compared with placebo disappeared within weeks after treatment ended, likely due to mutations conferring resistance also reducing bacterial fitness. In Norway, this study has been cited to justify more liberal amoxicillin prescribing for young children who refuse phenoxymethylpenicillin suspension because of its unpleasant taste [32].

The strong correlation between use of oral aminopenicillins and CRHI incidence in the present study (Figure 1A) suggests that cefotaxime resistance in *H. influenzae* does not reverse as rapidly as penicillin resistance in oral streptococci and supports the phenoxymethylpenicillin‐based Scandinavian approach. Furthermore, CRHI incidence in Norwegian hospitals tripled during the study period [11], despite declining selection pressure from oral aminopenicillins and third‐generation cephalosporins [10]. A likely explanation is that mutational beta‐lactam resistance impairs bacterial fitness to a lesser extent in *H. influenzae* . This is supported by an experimental study in which two of six resistant mutants selected under ampicillin exposure exhibited significantly increased fitness [17].

Antibiotic resistance evolves faster than new treatment options are developed [6, 26]. Effective antibiotics should therefore be regarded and managed as a non‐renewable resource. This study illustrates that choosing narrow‐spectrum agents when clinically appropriate increases the likelihood that broad‐spectrum alternatives remain effective when truly needed.

## Funding

This study was supported by a grant from the South‐Eastern Norway Regional Health Authority (15/00364‐75) to DS. The funding source played no role in the design and conduct of the study, collection, analysis, and interpretation of the data, or the preparation, review, or approval of the manuscript.

## Conflicts of Interest

The authors declare no conflicts of interest.

## Data Availability

The data that support the findings of this study are available through reference [11] and the sources described in the Materials and methods section.
